# *ADAM9* Genetic Variants and Their Role in Modulating Enzyme Activity in Diabetes and Metabolic Traits

**DOI:** 10.1155/jdr/5519447

**Published:** 2025-04-28

**Authors:** Hana Drobiova, Fahd Al-Mulla, Rabeah Al-Temaimi

**Affiliations:** ^1^Department of Pathology, College of Medicine, Kuwait University, Jabriya, Kuwait; ^2^Translational Medicine Department, Dasman Diabetes Institute, Dasman, Kuwait

**Keywords:** ADAM9, insulin resistance, metabolic syndrome, polymorphism, SNP, variants

## Abstract

A disintegrin and metalloproteinase Domain 9 (ADAM9) is a zinc-dependent proteinase involved in various biological processes. However, its role in the pathophysiology of metabolic syndrome remains unclear, and studies exploring the association between ADAM9 polymorphisms and metabolic traits are limited. In this study, we investigated the potential link between ADAM9 variants and metabolic syndrome traits in a cohort of adult participants from Kuwait. Using a genome-wide association study (GWAS), followed by a replication study, we identified two ADAM9 variants—ADAM9-E76K (rs61753672) and ADAM9-P750L (rs144750648)—that were associated with various metabolic traits. The replication phase confirmed the association of ADAM9-P750L with HbA1c levels and revealed new associations with systolic blood pressure, waist-to-hip ratio, fasting blood glucose, triglycerides, and cholesterol. Functional analysis showed that both variants exhibited reduced proteolytic activity, potentially contributing to the pathogenesis of Type 2 diabetes. These findings suggest that ADAM9 variants may play a significant role in metabolic health and diabetes risk.

## 1. Introduction

A disintegrin and metalloproteinases (ADAMs) are a family of secreted and transmembrane proteins that affect cellular phenotypes by their proteolytic activity to shed ectodomains of several protein classes, including growth factors, adhesion molecules, cytokines, and receptors and facilitate adhesion and migration by their interactions with integrins [1]. ADAMs are associated with several pathological processes in humans, including inflammation [2]; neurological disorders, namely, Alzheimer's disease [3, 4]; cardiovascular diseases [5]; and cancer [1, 6, 7]. However, research into their role in metabolic syndrome has not been completely elucidated.

Several ADAMs, including ADAM10, ADAM17, and ADAM19, were shown to play a role in insulin resistance and metabolic syndrome. For example, hyperglycaemia and insulin therapy increased ADAM10 serum concentration [8]. Increased expression and activity of ADAM17 were associated with insulin resistance and hypertension development, respectively [9, 10]. Moreover, increased ADAM17 expression in endothelial cells resulted in endothelial cell insulin resistance via cleavage of the insulin receptor extracellular domain [11, 12]. ADAM19 exhibits a strong correlation with metabolic syndrome traits, including body mass index (BMI), relative fat, homeostatic model assessment of insulin resistance (HOMA-IR), and triglycerides in humans [13].

ADAM9, a member of the ADAM family, has earned our attention for its potential role in metabolic syndrome and type 2 diabetes (T2D) development. Two ADAM9 isoforms are expressed, namely, the transmembrane (ADAM9-L) and secreted (ADAM9-S) isoforms, which are produced by alternative splicing of mRNA transcripts [14]. The transmembrane ADAM9-L is composed of a multidomain extracellular region, a single transmembrane domain, and a short cytoplasmic domain [15–17], while the secreted ADAM9-S lacks the transmembrane and cytoplasmic domains due to the excision of exon 12, and these are replaced by eight unique amino acids (LSLKFHAPF) that are absent in ADAM9-L. The extracellular region is composed of different domains including a prodomain, a metalloproteinase, a disintegrin, and cysteine-rich domains [14, 18, 19].

ADAM9-L is a Type I transmembrane glycoprotein that is involved in many biological functions [1, 14, 15, 20, 21] including heart development [22], neovascularization [23], wound healing [24], and macrophage fusion [25, 26]. Thus, it may contribute to the development of diabetic complications. Moreover, ADAM9 is widely expressed in human tissues, including the lung [27], colon [28], kidney [29], vascular smooth muscle [5, 30], nervous system [15, 31], reproductive system [32], and secretary organs [33]. Despite the global expression of ADAM9, knockout mice appeared to develop normally, were able to survive and reproduce, and did not display any abnormalities during both their developmental phase and adulthood. This suggests functional compensation or redundancy by other ADAMs [34].

*ADAM9* gene polymorphisms were shown to be associated with several diseases. Several mutations (rs786205085, rs137853040, rs137853041, and rs786205086) in the human *ADAM9* gene identified by autozygosity mapping are linked to recessively inherited early onset cone-rod dystrophy. These mutations result in the absence of a functional ADAM9 due to nonsense-mediated decay [35]. Moreover, homozygosity of a splice site mutation (rs786205151) in the *ADAM9* gene was observed in three affected members of a consanguineous Egyptian family with cone-rod dystrophy [36]. Based on these findings, a reanalysis of ADAM9-null mice revealed abnormalities in the gap between photoreceptor outer segments and the retinal pigment epithelium. By 20 months of age, ADAM9-null mice exhibited signs of progressive retinal degeneration [35]. Moreover, ADAM9 is involved in neurodegenerative diseases because it acts as an *α*-secretase that prevents the accumulation of amyloid beta (Ab) peptide. Polymorphisms (−542C/T rs10105311, −600A/C rs7840270, −963A/G rs6991968, and −1314T/C rs7006414) within the *ADAM9* promoter showed an association with Alzheimer's disease, with (−542C/−600A/−963G/−1314C) forming a protective haplotype and 963G/A-1314C showing higher transcriptional activity [37].

Limited research regarding the function of ADAM9 in metabolic syndrome indicates that during the development of the pancreas in both murine and human models, ADAM9 is confined to the insulin-producing b-cells, potentially participating in the cleavage of heparin-binding EGF-like growth factor (HB-EGF) [33, 38, 39]. Moreover, the intronic variant rs117316676 was associated with reduced levels of the diacylglycerol (DAG) palmitoyl-oleoyl-glycerol (16:0/18:1) [40], potentially offering protection against the onset of insulin resistance and T2D. Although a recent study [41] reported that downregulation of ADAM9-S ameliorated insulin resistance in gestational diabetes by modulating the miR-375/FPR2 axis and inactivation of the MAPK pathway, the function of ADAM9 in metabolic syndrome remains unclear, and investigations examining its polymorphic association with metabolic syndrome are limited. Moreover, earlier research has identified several genetic variants associated with metabolic traits including obesity, insulin resistance, and dyslipidaemia, especially in populations of European and East Asian descents. Yet, only few studies have focused on populations from the Middle East, especially the Kuwaiti population, where environmental and genetic factors differ significantly [42–46]. We believe that the two novel *ADAM9* variants, namely, ADAM9-E76K rs61753672 and ADAM9-P750L rs144750648, identified in this study provide a novel insight into the genetic framework of metabolic traits within Kuwait's population. Moreover, our functional assessment of these variants suggests that they may have a potentially significant role in the development of metabolic syndrome and T2D in Kuwait's population. Thus, this study enhances our understanding of metabolic traits and emphasizes the need for further population-specific studies, which may provide novel and personalized diagnostic and therapeutic strategies based on the unique genetic framework of this specific population and other populations with similar environmental and genetic characteristics.

Our study has identified two novel ADAM9 variants, namely, ADAM9-E76K rs61753672 and ADAM9-P750L rs144750648, to be associated with metabolic syndrome traits. Therefore, to better understand the pathophysiology of metabolic syndrome, we explored *ADAM9* missense variants' associations with metabolic syndrome traits in a cohort of adults in Kuwait and investigated the functional effects of these polymorphisms.

## 2. Materials and Methods

### 2.1. Study Participants of Discovery and Replication Phases

The discovery phase was conducted as previously published [45, 46] using a genome-wide association study (GWAS) as part of the Kuwait Obesity Genome Project (KOGP) conducted at Dasman Diabetes Institute (DDI) in Kuwait. A total of 1298 randomly selected participants, including individuals of Arab origin, were recruited from the six governorates of Kuwait. The replication phase cohort was composed of a total of 859 participants from the Kuwait Diabetes Epidemiology Project (KDEP) conducted at DDI. The participants were randomly chosen to represent the adult population of Kuwait by a stratified random selection method from a computerized database maintained by the Public Authority of Civil Information, which contains personal data on Kuwaiti nationals and expatriates [47]. The inclusion criteria for the discovery phase included confirmation of the Arab ethnicity by a questionnaire about parental lineage, which considered parental lineages up to the third generation. On the other hand, the replication phase allowed the inclusion of any ethnicity. Exclusion criteria for both the discovery and the replication phases included age below 18 years, the presence of chronic or serious diseases such as complications of diabetes and hypertension, mental illness, or cognitive impairment. These patients were excluded to avoid additional confounders such as hormonal imbalance and therapeutic responses, for which adjustments would need to be carried out for genetic association modelling. Moreover, pregnant women and patients undergoing weight reduction medication/surgery or fitness programs were also excluded [45].

After verification that participants fasted overnight, they signed a consent form, and blood samples were collected. At the same time, their vital signs were recorded, and the nationality and ethnicity of each participant were verified using a detailed questionnaire. The participants' medications for diabetes, hypertension, and cholesterol lowering were noted and used in association statistics rectification process. Moreover, verification of other illnesses, such as diabetes and cardiovascular complications, was also noted. All procedures were conducted following the guidelines of the Institutional Ethical Review Committee according to the guidelines of the Declaration of Helsinki and the US Federal Policy for the Protection of Human Subjects (ERB/ERC numbers: RA HM 2010-005 for KOGP and RA HM 2010-004 for KDEP). The clinical assays conducted for trait outcome measurements were carried out at a College of American Pathologists (CAP)–accredited laboratory at DDI [46].

### 2.2. ADAM9 Variants Genotyping

The *ADAM9* genotypes of participants in the discovery phase were explored using the Illumina HumanOmniExpress and the iScan system (Illumina) utilizing the Infinium HD Assay Ultra genotyping assay method. The intensity values of all genotyped samples were combined, and genotype calling was performed. To shortlist the genetic associations, naïve *p* values were used, and association tests were corrected for various confounders such as age, sex, stratification, medication, and obesity/diabetes status. The two study variants exhibited suggestive GWAS *p* values and deserved to be examined in an independent cohort.

The replication study was conducted by targeted genotyping of the selected *ADAM9* single-nucleotide polymorphisms (SNPs) including ADAM9-E76K rs61753672 and ADAM9-P750L rs144750648. SNP genotyping was performed by the TaqMan SNP genotyping assay (Applied Biosystems, Massachusetts, United States) using the QuantStudio 5 real-time polymerase chain reaction (PCR) system (Applied Biosystems, Massachusetts, United States). The PCR was conducted using a 20 *μ*L reaction containing 20 ng of genomic DNA, 10 *μ*L of 2X TaqMan Genotyping master mix, 1 *μ*L of 20X TaqMan SNP genotyping assay, and nuclease-free water to make up the volume to 20 *μ*L. The thermal cycling conditions included a pre-PCR read at 60°C for 1 min followed by the polymerase activation at 95°C for 10 min, 40 cycles of denaturation at 95°C for 15 s, and annealing/extension at 60°C for 1 min. Finally, a post-PCR read was performed at 60°C for 1 min. Sanger sequencing of PCR products of selected cases of homozygotes and heterozygotes was performed to confirm the obtained genotypes. The sequencing reactions were conducted using the BigDye terminator cycle sequencing kit v.3.1 (Applied Biosystems, Massachusetts, United States) according to the manufacturer's instructions on an ABI PRISM 3730xl genetic analyser (Applied Biosystems, Massachusetts, United States).

### 2.3. Structural Analysis

The identified *ADAM9* variants were mapped, modelled, and analysed using PyMOL modelling software (the PyMOL Molecular Graphics System, Version 3.0 Schrödinger, LLC). The effect of genetic variants on ADAM9 stability and flexibility was studied using the DynaMut web server. Both analyses used ADAM9 predicted crystal structure (accession No. Q13443). The predicted structure was developed using homology modelling machine learning with various degrees of prediction confidence (UniProt).

### 2.4. DNA Constructs and Mutagenesis

The human-tagged ORF clone plasmid encoding *ADAM9* was purchased from OriGene Technologies (RC222453, Rockville, Maryland, United States). Site-directed mutagenesis of SNPs was conducted using the QuikChange II site-directed mutagenesis kit (Agilent Technologies, Santa Clara, California, United States) according to the manufacturer's instructions. Successful mutations were verified by Sanger sequencing. The sequencing reactions were conducted using the BigDye terminator cycle sequencing kit (Applied Biosystems, Massachusetts, United States) according to the manufacturer's instructions on an ABI PRISM 3730xl genetic analyser (Applied Biosystems, Massachusetts, United States). The primers used to generate the mutations and to verify them by sequencing are listed in Table 1.

### 2.5. Cell Culture, DNA Transfections, and Western Blotting

HEK293 cells were purchased from ATCC (CRL-1573, Manassas, Virginia, United States). The cells were grown in Dulbecco's modified Eagle's medium (DMEM) (Invitrogen Corporation, Carlsbad, California, United States) supplemented with 10% fetal bovine serum (FBS) (Invitrogen Corporation) and 100 U/mL of penicillin and 100 *μ*g/mL of streptomycin (Gibco, Carlsbad, California, United States) and were incubated at 37°C in a humidified atmosphere containing 5% CO_2_. The cells (2 × 10^5^ or 5 × 10^4^) were seeded into 6-well or 96-well plates, respectively, for 24 h prior to transfection. HEK293 cells in 6-well or 96-well plates were transfected with 2 *μ*g or 200 ng, respectively, of wild-type (WT) or mutant constructs using lipofectamine 3000 reagent (DNA:lipofectamine ratio 1:2) (Invitrogen, Massachusetts, United States) in an antibiotic-free medium. Twenty four hours after transfection, the medium was changed to complete DMEM media, in which cells were grown for another 24 h, before being harvested for protein analysis. Overexpression of the WT and mutant proteins was confirmed using western blots.

Western blot analysis was performed by harvesting and lysing cells in an extraction buffer containing 0.5% NP-40 and 2% CHAPS in phosphate-buffered saline (PBS) (Gibco, Carlsbad, California, United States). The lysates were centrifuged at 4°C for 15 min at 13,000 rpm. The protein concentration in the supernatant was quantified using the Bradford assay (Thermo Fischer Scientific, Driesch, Germany). Then, 5 *μ*g of total protein was resolved on 8% denaturing polyacrylamide gel and transferred to a nitrocellulose membrane (EMD Millipore Corporation, Massachusetts, United States). Membranes were blocked using 5% fat-free milk and blotted with the corresponding primary and horseradish peroxidase (HRP)–linked secondary antibodies. The primary antibodies used were b-actin (ab3700, Abcam, Massachusetts, United States), ADAM9 (ab186833, Abcam, Massachusetts, United States), and FLAG-tag (F1804, Sigma-Aldrich, Missouri, United States). Chemiluminescent detection of HRP activity was achieved using a chemiluminescent substrate (Thermo Scientific, Massachusetts, United States) according to the manufacturer's instructions, and the membranes were visualized using a ChemiDoc Imaging System (Bio-Rad, Hercules, California, United States) and analysed by Image Lab software.

### 2.6. Enzyme Kinetic Activity of WT ADAM and Its Variants

The calibration fluorescent peptide (5⁣′-FAM-Pro-Leu-Arg-Arg-Thr-Leu-Ser-Val-Ala-Ala-OH) was purchased from CPC Scientific (Sunnyvale, California, United States), while the ADAM9 fluorogenic substrate PEPDAB010 (Dabcyl-SPLAQAVRSSK[5-FAM]-NH2) peptide was purchased from BioZyme Inc. (St. Joseph, Missouri, United States). A fluorescent calibration curve was generated using different concentrations (0–10 mM) of the calibration fluorogenic peptide. Then, cleavage assays of PEPDAB010 were performed in 96-well plates using 10 *μ*g of the total protein extracts from cells transfected with WT or mutant ADAM9 in a reaction buffer containing 1 mM CaCl_2_, 10 mM NaCl, 20 mM Tris pH 8.0, and 0.5% Brij-35. As negative controls, protein extracts from untransfected cells or cells transfected with a GFP-expressing plasmid were used. Reactions were conducted at 37°C, and fluorescence emission was measured for a total of 3 h at 5-min intervals using a multimode microplate reader (Synergy H4 Hybrid, BioTek Instruments, Vermont, United States) with excitation and emission wavelengths of 485 and 530 nm, respectively. Enzyme activities were normalized based on western blot analysis, and the maximum velocity (*V*_max_) and the Michaelis–Menten constant (*K*_m_) were determined using the Lineweaver–Burk plot enzyme kinetic model [47].

### 2.7. Statistical Analysis

Clinical and demographic data were analysed using Student's *t*-test or the nonparametric Mann–Whitney test. Hardy–Weinberg equilibrium was assessed in the healthy control cohort for the two variants using expected allele frequencies by the *χ*^2^-test. To determine the allele effect size (*β* coefficient), linear regression analysis was performed. ADAM9 activity assays were done using two biological replicates, and data are presented as mean ± standard deviation. Biological replicates mean that data were obtained using different transfection reactions of cultured cells. Significance was assessed using an unpaired two-tailed *t*-test. *p* values ≤ 0.05 were considered statistically significant. Statistical analyses were performed using SPSS v.25 (IBM, New York, United States).

## 3. Results

### 3.1. ADAM9 Variants Identified in the Discovery Phase

**Figure 1:**
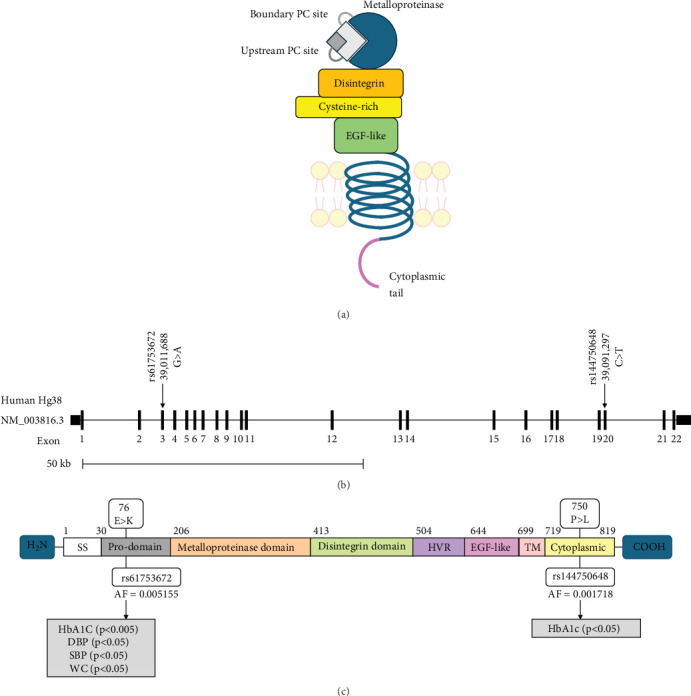
ADAM9 characteristics (a) and the location metabolic syndrome associated with SNPs identified by GWAS on ADAM9-L gene (b) and protein (c). Shaded boxes indicate the association of the variant with increased values of the trait(s). DBP: diastolic blood pressure; HbA1c: haemoglobin A1c; SBP: systolic blood pressure; WC: waist circumference (BMI adjusted).

A discovery GWAS conducted as a part of the KOGP study at DDI included 1298 Arab participants with the demographic and clinical data of healthy controls and T2D patients (Table 2). This study revealed a significant (*p* < 0.05) association of two *ADAM9* variants, namely, ADAM9-E76K rs61753672 and ADAM9-P750L rs144750648, with several traits of the metabolic syndrome (Table 3), including haemoglobin A1c, waist circumference, and systolic and diastolic blood pressure. The locations of these variants on *ADAM9* gene and protein are illustrated in Figure 1. Therefore, these variants were considered for replication using a different cohort of participants including adult residents from Kuwait who were part of KDEP.

### 3.2. Replication Phase

To confirm the findings of the discovery phase, *ADAM9* variants' association with metabolic syndrome traits was studied in a different cohort including healthy controls (*n* = 409) and type 2 diabetic patients (*n* = 450) from the KDEP study. This cohort included adults of different nationalities from Kuwait. The demographic and clinical data of the replication cohort participants are summarized in Table 4. The minor allele frequencies (MAFs) of ADAM9-E76K rs61753672 and ADAM9-P750L rs144750648 variants (0.017 and 0.097, respectively) were higher in the replication cohort compared to those detected in the discovery phase (0.005 and 0.002, respectively) as well as compared to those reported in gnomAD database (0.006109 and 0.0002794, respectively) (gnomAD v2.1.1). These differences in MAFs could be attributed to population stratification (Arab ethnicity in the discovery cohort vs. multiple ethnicities in the replication cohort) and difference in genotyping technique used (while the variant in the discovery cohort was imputed based on markers genotyped using bead array chip, the variant in the replication cohort was target genotyped). ADAM9-E76K rs61753672 variant was in Hardy–Weinberg equilibrium in the healthy cohort (*p* > 0.05), but ADAM9-P750L rs144750648 was not in Hardy–Weinberg equilibrium in the healthy cohort (*p* < 0.05). Associations of *ADAM9*-E76K rs61753672 detected in the total discovery cohort were not confirmed in the total replication cohort. However, in the replication phase, ADAM9-E76K rs61753672 was associated with high HDL levels within the type 2 diabetic cohort (*β* = 0.078; 95% CI: 0.018–0.137; *p* = 0.01). These differences between the two cohorts can be attributed to differences in sample sizes (discovery cohort [1298 participants] vs. replication cohort [589 participants]) and the ethnic composition of the two cohorts, with the discovery cohort solely composed of Arab individuals while the replication cohort including individuals of different ethnicities. The replication cohort confirmed the association of ADAM9-P750L rs144750648 with HbA1c (*β* = 0.027; 95% CI: 0.013–0.041; *p* < 0.001) and revealed associations with other metabolic syndrome traits, including SBP (*β* = 0.002; 95% CI: 0.001–0.003; *p* = 0.002), BMI (*β* = 0.005; 95% CI: 0–0.009; *p* = 0.04), waist:hip ratio (*β* = 0.793; 95% CI: 0.498–1.089; *p* < 0.001), FBG (*β* = 0.019; 95% CI: 0.010–0.028; *p* < 0.001), TGL (*β* = 0.044; 95% CI: 0.020–0.068; *p* < 0.001), and TC:HDL ratio (*β* = 0.026; 95% CI: 0.013–0.040; *p* < 0.001) (Table 5). The associations of ADAM9-750 rs144750648 with waist:hip ratio, TGL, and TC:HDL ratio were sustained when tested in healthy and T2D cohorts separately (Table 5), whereas the association of ADAM9-P750L rs144750648 with HbA1c and SBP was only detected in the T2D cohort (Table 5). In contrast, BMI association was detected in the healthy cohort (*p* = 0.020) but not in the T2D cohort (*p* = 0.258). Moreover, the association of ADAM9-P750L rs144750648 variant with HbA1c, FBG, and TC:HDL ratio was sustained after adjustment for confounding factors including age, gender, and BMI.

### 3.3. Effect of ADAM9 Variants on Protein Structure and Stability

**Figure 2:**
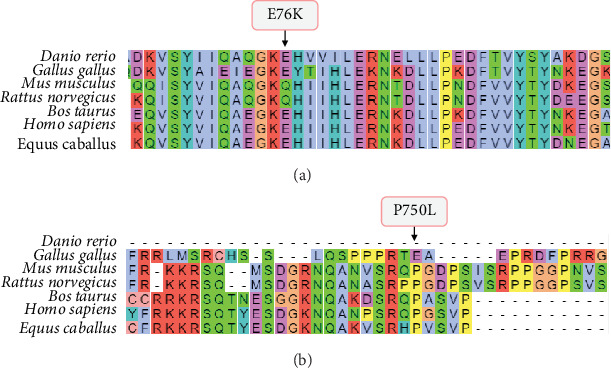
Sequence alignment of ADAM9 prodomain (a) and cytoplasmic (b) domains indicating the position of the missense mutations E76K and P750L, respectively.

The variants identified within the *ADAM9* gene, including ADAM9-E76K rs61753672 and ADAM9-P750L rs144750648, are missense variants that result in the replacement of amino acids within the prodomain (E76K) and the cytoplasmic (P750L) domain, respectively (Figure 1). The two identified variants 76K and 750L involve amino acid residues that are highly conserved across species (Figure 2).

**Figure 3:**
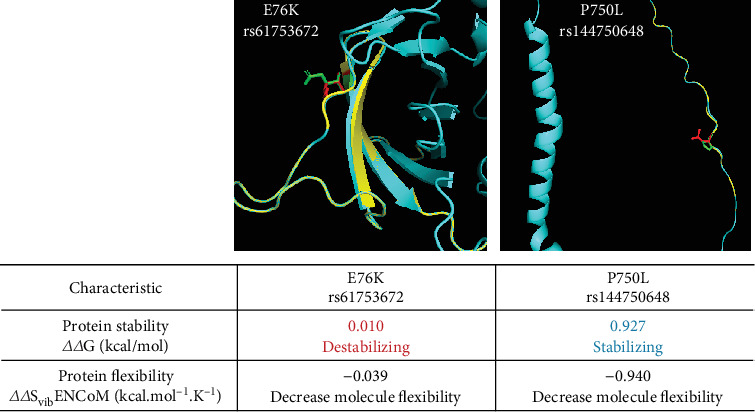
ADAM9 variants (yellow) are mapped to the structure of the protein (blue). DynaMut analysis showed that 76K variant destabilizes the protein and reduces its flexibility, while the P750L variant stabilizes the protein and reduces its flexibility.

To predict the effect of these variants on ADAM9 protein structure and its stability and flexibility, the variants were analysed using PyMOL and DynaMut software (Figure 3). ADAM9-76K rs61753672 variant was predicted to destabilize the protein and reduce its flexibility, while ADAM9-750L rs144750648 variant stabilizes the protein and reduces its flexibility.

### 3.4. Enzyme Activity and Kinetic Parameters of the ADAM9 Variants

**Figure 4:**
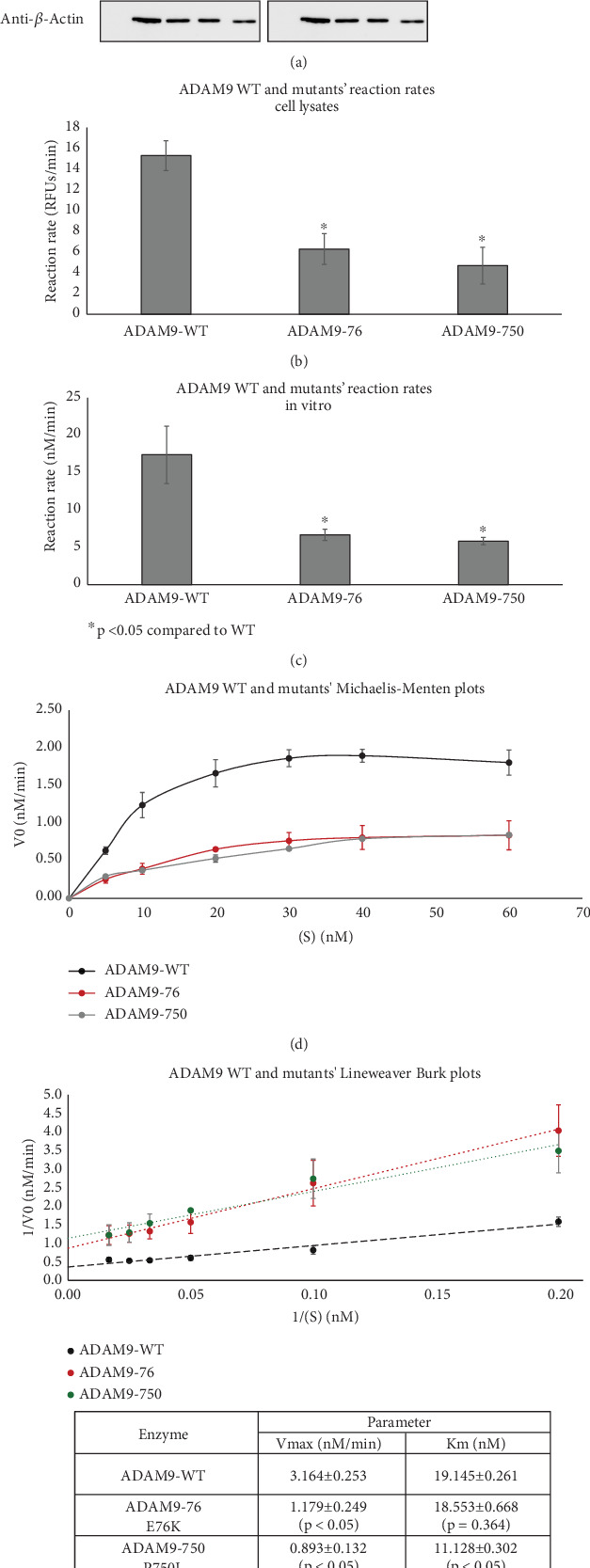
The effect of ADAM9 variants on enzyme kinetic parameters. (a) Western blot analysis of ADAM9 variants (ADAM9-76 and ADAM9-750) using anti-ADAM9 and anti-FLAG antibodies, and *β*-actin as a loading control. ADAM9-Flag tagged constructs of WT and variants were transfected in HEK293 cells. Control cells were transfected with empty vectors. The immunoblots confirm the overexpression of ADAM9 WT and variants in the cells. (b) ADAM9 enzymatic activity was measured using cell lysate containing 10 μg of total protein and 40 μM fluorogenic substrate PEPDAB010, at excitation and emission wavelengths of 485 and 530 nm. The fluorescence was detected using Synergy H4 reader and normalized to a reaction containing the substrate alone. Enzyme activity was determined using a calibration standard curve and was normalized to western blots. Two-tailed unpaired Student's *t*-test was used to determine significance. Experiments were done in duplicates using two different cell extracts. Values with *p* < 0.05 were considered significant. (c) ADAM9 enzyme activity was measured in intact cells, using the same approach as described in (b). Enzyme kinetics were studied by drawing the Michaelis–Menten plot (d) and Lineweaver–Burk plot (e) to calculate *V*_max_ and *K*_m_.

As previously mentioned, the studied *ADAM9* missense variants are located within the prodomain and the cytoplasmic domains. To examine the functional significance of these *ADAM9* variants on its proteolytic activity, *ADAM9* WT and *ADAM9* (76K and 750L) variants were generated using site-directed mutagenesis assay and were separately expressed in HEK293 cells. Western blot analysis confirmed overexpression of proteins in HEK293 cell lysates (Figure 4). Enzymatic assay conducted using the fluorogenic substrate PEPDAB010 showed that all ADAM9 variants studied exhibit a significant reduction in the catalytic activity compared to WT ADAM9 (76K 58.1 ± 12.9% and 750L 68.5 ± 13.6%; *p* < 0.05; Figure 4). To rule out possible interference with mutants' activity within cells, HEK293 cells grown on a 96-well black plate were separately transfected with *ADAM9* WT and *ADAM9* variants as described above. The activity of the enzyme was measured using the cells directly without lysing, enabling the measurement of the activity of the enzymes that were present at the cell surface. The results showed similar reductions in proteolytic activity (76K 61.7 ± 4.4% and 750L 66.5 ± 1.0; Figure 4).

To further evaluate the effect of *ADAM9* variants on its proteolytic activity, kinetic parameters, including *V*_max_ and *K*_m_, were determined. As illustrated in Figure 4d,e, ADAM9 WT and the studied variants followed the Michaelis–Menten equation, and the Lineweaver–Burk plot was used to calculate *V*_max_ and *K*_m_ values. The *V*_max_ value of 76K and 750L variants was significantly reduced by 62.7% and 71.8%, respectively, compared to ADAM9 WT (Figure 4e). The *K*_m_ value of 76K was slightly reduced by 3.9% compared to WT, whereas the *K*_m_ value of the 750L variant was significantly reduced by 41.9%, compared to ADAM9 WT.

## 4. Discussion

ADAM9 is known to be involved in many physiological processes. However, ADAM9's role in metabolic syndrome is still to be elucidated, and research into *ADAM9* variants' association with metabolic syndrome traits is limited. Herein, using GWAS, we have identified two *ADAM9* variants, namely, rs61753672 and rs144750648 that exhibit significant association with metabolic syndrome quantitative traits, including HbA1c, waist circumference, and hypertension. These observations were confirmed in our replication study for the rs144750648 variant.

The higher MAFs of rs61753672 and rs144750648 variants in our replication cohort compared to those reported in our discovery cohort and gnomAD may have resulted from natural selection or consanguineous marriages and may partially explain the prevalence of metabolic syndrome and T2D in Kuwait's population [48]. The association of the rs61753672 variant with different metabolic traits reported in our GWAS was not confirmed in our replication cohort. This may be explained by the small size effect and the ethnic variation of the replication cohort compared to that in the discovery cohort. In addition, the rs61753672 variant is associated with HDL in the T2D replication cohort alone, suggesting it plays a role in HDL related cardiovascular complications [5]. On the other hand, the association of rs144750648 with HbA1c revealed in the discovery cohort was confirmed, and associations with other traits were revealed in the replication cohort. These differences may be related to the different genetic backgrounds of the discovery and replication cohorts. To the best of our knowledge, this is the first study that reports these *ADAM9* variants as risk factors for metabolic syndrome, although some studies allude to an association between *ADAM9* and diabetic complications. For example, increased expression of ADAM9 in diabetic macrophages was found to impair apoptotic cardiomyocytes' efferocytosis [49] and was also involved in phagocytosis impairment of photoreceptor outer segments in retinal pigment epithelial cells [50].

As mentioned above, the studied *ADAM9* variants (rs61753672 and rs144750648) are missense variants that lead to amino acid changes in critical domains within the enzyme. In addition, they are highly conserved among different species. Therefore, it is expected that these mutations may affect protein structure and function. We found that E76K and P750L mutants significantly reduced the proteolytic activity of ADAM9, but only P750L caused a significant reduction in *K*_m_, hence increasing its affinity to the substrate PEPDAB010. These changes may have resulted from the mutation affecting protein stability and flexibility, which was decreased in the case of the prodomain mutant E76K. The proximity of this variant to the R56 furin cleavage site may reduce furin access and prevent the cleavage of the prodomain [51], thus inhibiting the proteolytic activity of the enzyme [52]. Moreover, the P750L mutation within the cytoplasmic tail may potentially affect the ability of the protein to bind SH3 domain–containing proteins, the majority of which are sorting- and endocytosis-associated adaptor proteins or kinases [14]. However, based on the results shown in viable cells, this possibility was eliminated as the proteins behaved the same as in the cell lysate. Therefore, possibly an allosteric mechanism may be involved in the reduced activity of this mutant, especially since it was associated with reduced protein flexibility.

Recent research indicates that ADAM9 has both proteolytic activity and cell adhesion properties [17]. While several potential substrates for ADAM9 have been identified, detailed research on the signalling pathways associated with ADAM9 in the context of disease is still lacking. The putative ADAM9 substrates include transmembrane proteins such as pro-HB-EGF, pro-EGF, amyloid precursor protein, kit ligand, necrosis factor *α* (TNF-*α*), p75 neurotrophic receptor, insulin B-chain, delta-like Ligand 1, IGFBP-5, ADAM10, collagen XVII, laminin, and FGF Receptor 2 IIIb [17, 52–58].

Based on our reported findings, we provide the following mutant ADAM9 potential role in the context of T2D pathogenesis. The main role of pancreatic b-cells is to produce and release insulin in a regulated manner to maintain blood glucose levels within normal ranges [59, 60]. When peripheral tissues become resistant to insulin, the pancreas responds by increasing insulin production and secretion to compensate [61]. However, if this compensatory mechanism fails, T2D develops. The adaptive response of the b-cells includes both increased insulin secretion and an expansion of b-cell mass [62]. Despite the importance of this mechanism for the maintenance of normoglycemia in response to insulin resistance and hence preventing T2D development [63]; the mechanism is not elucidated yet. However, research shows that as the pancreas develops, ADAM9 becomes localized specifically to the insulin-producing cells in the islets of Langerhans [39], where ADAM9 can cleave pro-HB-EGF, influencing various aspects of pancreatic development through the EGFR pathway. EGFRs are highly expressed in both the endocrine pancreas and the primitive duct cells [64], and mice lacking EGFR have abnormal pancreatic islets [65]. The activated HB-EGF in duct cells may promote b-cell differentiation from duct cells and the proliferation of pre-existing b-cells, thus improving glucose tolerance via increasing b-cell mass [66]. The potential mitogenic role of HB-EGF through EGFR and mTOR is supported by the increased expression of HB-EGF in islets of insulin-resistant rats and its potential to activate mTOR and stimulate the proliferation of MIN6 cells and dispersed rat and human islets [67, 68]. Notably, HB-EGF can also activate the mTOR pathway in human islets in a PI3K-dependent manner [67]. In addition to HB-EGF, the pancreas also produces EGF; however, its circulating levels along with the EGFRs are reduced in diabetic animals [69–71]. B-cell exposure to EGF induces insulin secretion in a glucose-dependent manner [71]. Moreover, EGFR was demonstrated to be crucial for b-cell maturation and signalling [39, 72], because EGFR knockout pancreas exhibits impaired cell migration and differentiation. HB-EGF is produced as a membrane-anchored precursor (pro-HB-EGF) that is processed to release the soluble form of HB-EGF [66]. The release of the soluble active forms of both EGF and HB-EGF is mediated by ADAMs including ADAM9 [17, 73]. Therefore, the reduced ADAM9 activity of our variants may prevent the ability of the pancreas to sustain sufficient insulin production in response to peripheral insulin resistance leading to T2DM. Another mechanism of insulin resistance in obesity and T2D, particularly in skeletal muscles, involves extracellular matrix (ECM) remodelling [74]. ECM remodelling is induced by increasing ECM1 gene expression and collagen (mainly Types I and III) accumulation as demonstrated in healthy individuals in response to an increase in plasma-free fatty acid concentrations [75, 76] and occurs in obese individuals and individuals with metabolic disorders such as hyperglycaemia, hyperinsulinemia, and dyslipidaemia. It is proposed that ECM remodelling forms a mechanical barrier impeding insulin and glucose transport in the muscles or it may impair communication of integrin signalling pathways, which may inhibit insulin signalling [74]. In contrast, physical exercise improves insulin sensitivity in muscles because it induces the expression and activates matrix metalloproteases (MMPs) including MMP2, MMP9, and ADAMTS1 [77] facilitating normal ECM remodelling [78] by degrading ECM proteins [74]. ADAM9-deficient mice exhibit upregulation of collagen Types I and V and downregulation of MMPs [79, 80]. Therefore, the reduced activity of ADAM9 observed in our variants may contribute to insulin resistance and T2DM development via these ECM alterations. However, functional studies involving in vitro and in vivo models are necessary to explore the role of these ADAM9 variants in metabolic syndrome.

Moreover, ADAM9 substrates including tumour TNF-*α*, p75 neurotrophin receptor, and EphB4 were found to regulate cellular aspects related to metabolic syndrome and insulin resistance. TNF-*α* plays an important role in insulin resistance development through affecting key elements in the insulin signalling pathway, such as AKT phosphorylation [81], and owing to its potential to promote inflammation [82]. Also, chronic exposure to TNF-*α* may cause global alterations in the proteome and phosphoproteome leading to insulin resistance [83]. The p75 neurotrophin receptor (p75NTR) was shown to decrease insulin-stimulated glucose transport in adipocytes through its interaction with Rab5 and Rab31 in normal chow-fed mice. In contrast, the transplantation of p75NTR-null white adipose tissue into WT mice-fed high-fat diet prevented weight gain and insulin resistance [84, 85]. Interestingly, EphB4, a tyrosine kinase receptor, may bind directly to the insulin receptor facilitating its clathrin-mediated endocytosis and degradation by lysosomes [86, 87]. Therefore, the reduced ADAM9 proteolytic activity variant reported here may increase the availability of the mentioned substrates leading to the development of insulin resistance.

Despite the limitation that our study included a moderate sample size and participants of different genetic backgrounds in the discovery and replication cohorts, we were able to identify rare *ADAM9* variants and confirm their associations with T2D and metabolic syndrome traits. Yet, future studies including a wider range of ethnic and geographical populations would enhance validation and provide stronger evidence for their general applicability. Moreover, the reduced proteolytic activity of these variants suggests the high potential for a true functional association with T2D pathophysiology and may provide a novel target for its therapeutic treatment. However, further research is needed to validate, both in vitro and in vivo, the pathogenic role of ADAM9 in insulin resistance and T2D.

## 5. Conclusion

ADAM9 is a metalloproteinase enzyme that plays a role in many physiological functions. This study provides evidence for the potential role of this enzyme in the pathophysiology of T2D/metabolic syndrome. It reveals that ADAM9 variants (76K and 750L) are potentially associated with metabolic syndrome traits. Mechanistically, the reduced proteinase activity may impair insulin production in b-cells and may cause insulin resistance in muscles and adipose tissue. However, further research is required to elucidate the exact mechanism.

## Figures and Tables

**Table 1:** Primers used for *ADAM9* site-directed mutagenesis and its verification by sequencing.

**Gene-aa-(base change)**	**SNP**	**Primer name**	**Primer sequence**
ADAM9-76 (G>A)	rs61753672	hADAM9-76-F	TTATTCAGG**CAGAAG**GAAAA**A**AGCATATTATTCACTTGGAA
		hADAM9-76-R	TTCCAAGTGAATAATATGCT**T**TTTTCCTTCTGCCTGAATAA
		hADAM9-76-SeqF	CGGGAATTCGTCGACTGGAT
		hADAM9-76-SeqR	CTGAGTCCAAAACAGTCGCT

ADAM9-750 (C>T)	rs144750648	hADAM9-750-F	AAGCAAACCCT**TCTAGG**CAGC**T**GGGGAGTGTTCCTCGACAT
		hADAM9-750-R	ATGTCGAGGAACACTCCCC**A**GCTGCCTAGAAGGGTTTGCTT
		hADAM9-750-SeqF	ATTGAGGGACGGACTTCTGG
		hADAM9-750-SeqR	CTCTCGTCGCTCTCCATCTC

*Note:* Bold-underlined nucleotides represent restriction sites (either modified or not) to enable the identification of mutant plasmids. Only bold font represents sites of mutagenesis.

**Table 2:** Demographic and clinical characteristics of healthy control and Type 2 diabetic patients included in the discovery phase. All quantitative variables are represented as mean ± standard deviation unless otherwise indicated.

**Criteria**	**Healthy controls (** **n** = 697**)**	**Type 2 diabetics (** **n** = 601**)**	**p** ** value**
Gender, *n* (%)			0.7
Male	350 (50)	308 (51)	
Female	347 (50)	293 (49)	
Age, years	41 ± 13	54 ± 11	< 0.001
Body mass index	31 ± 8	34 ± 7	< 0.001
Wasit:hip ratio	0.43 ± 1.25	0.50 ± 1.19	0.2748
FBG, mmol/L	5.1 ± 2.0	9.5 ± 4.5	< 0.001
HbA1c, %	5.65 ± 0.86	8.21 ± 2.04	< 0.001
Total cholesterol, mmol/L	5.07 ± 1.06	5.02 ± 1.21	0.15
Triglycerides, mmol/L	1.53 ± 1.05	1.92 ± 1.35	< 0.001
HDL, mmol/L	1.16 ± 0.40	1.09 ± 0.36	0.002
LDL, mmol/L	3.29 ± 0.97	3.07 ± 1.07	< 0.001
Total cholesterol:HDL ratio	4.84 ± 1.87	5.06 ± 2.12	0.10
SBP, mmHg	123 ± 16	133 ± 18	< 0.001
DBP, mmHg	77 ± 11	79 ± 10	< 0.001

Abbreviations: DBP: diastolic blood pressure; FBG: fasting blood glucose; HbA1c: glycated haemoglobin; HDL: high-density lipoprotein; LDL: low-density lipoprotein; SBP: systolic blood pressure.

**Table 3:** Missense (exonic) ADAM9 variants associated with metabolic syndrome traits identified in the discovery phase.

**Variant ID**	**Minor allele frequency**	**Amino acid change**	**Traits associated with variant**	**β**	**p** ** value**
ADAM9-76 rs61753672	0.005	E76K	HbA1c	2.53	0.001
			WC	1.11	0.044
			DBP	1.33	0.026
			SBP	1.24	0.037

ADAM9-750 rs144750648	0.002	P750L	HbA1c	4.98	0.016

Abbreviations: DBP: diastolic blood pressure; HbA1c: glycated haemoglobin; SBP: systolic blood pressure; WC: waist circumference.

**Table 4:** Demographics and clinical characteristics of healthy control and Type 2 diabetic patients included in the replication phase. All quantitative variables are represented as mean ± standard deviation unless otherwise indicated.

**Criteria**	**Healthy controls (** **n** = 409**)**	**Type 2 diabetics (** **n** = 450**)**	**p** ** value**
Gender, *n* (%)			0.014
Male	232 (56.7)	292 (64.9)	
Female	177 (43.3)	158 (35.1)	
Age, years	40.41 ± 10.70	45.79 ± 10.453	< 0.001
Body mass index	28.78 ± 5.98	29.79 ± 5.656	0.005
Wasit:hip ratio	0.871 ± 0.092	0.922 ± 0.065	< 0.001
FBG, mmol/L	4.717 ± 0.383	6.708 ± 2.764	< 0.001
HbA1c, %	5.057 ± 0.535	6.594 ± 1.648	< 0.001
Total cholesterol, mmol/L	5.039 ± 0.966	5.291 ± 1.014	0.001
Triglycerides, mmol/L	1.345 ± 0.750	1.732 ± 0.970	< 0.001
HDL, mmol/L	1.232 ± 0.378	1.110 ± 0.300	< 0.001
LDL, mmol/L	3.226 ± 0.881	3.443 ± 0.927	0.002
Total cholesterol:HDL ratio	4.406 ± 1.428	5.060 ± 1.633	< 0.001
SBP, mmHg	124.90 ± 18.342	134.24 ± 18.90	< 0.001
DBP, mmHg	77.28 ± 11.875	81.23 ± 11.79	< 0.001

Abbreviations: DBP: diastolic blood pressure; FBG: fasting blood glucose; HbA1c: glycated haemoglobin; HDL: high-density lipoprotein; LDL: low-density lipoprotein; SBP: systolic blood pressure.

**Table 5:** *ADAM9* variants associated with various metabolic biomarkers in the replication phase when considering the total population sample or healthy controls or diabetic patients alone. *p* values of significance (*p* < 0.05) are shown in bold font.

**Variant**	**Trait**	**Replication phase associations**			
		**Cohort**	**β**	**95% CI**	**p** ** value**
ADAM9-76 rs61753672	HbA1c	Complete	−0.007	−0.016 to 0.002	0.117
		Healthy controls	0.021	−0.022 to −0.065	0.339
		Diabetic patients	−0.010	−0.020 to 0.001	0.084
	DBP	Complete	0.001	0 to 0.002	0.095
		Healthy controls	0.001	−0.001 to 0.003	0.512
		Diabetic patients	0.001	0 to 0.003	0.080
	SBP	Complete	0	0 to 0.001	0.362
		Healthy controls	0.001	−0.001 to 0.002	0.239
		Diabetic patients	0	−0.001 to 0.001	0.632
	WC	Complete	0	−0.001 to 0.002	0.378
		Healthy controls	0.002	0 to 0.004	**0.050**
		Diabetic patients	6.575 × 10^−5^	−0.001 to 0.001	0.926
	HDL	Complete	0.028	−0.014 to 0.070	0.197
		Healthy controls	−0.029	−0.091 to 0.033	0.355
		Diabetic patients	0.078	0.018 to 0.137	**0.010**

ADAM9-P750L rs144750648	HbA1c	Complete	0.027	0.013 to 0.041	**< 0.001**
		Healthy controls	0.020	−0.033 to 0.073	0.450
		Diabetic patients	0.022	0.004 to 0.041	**0.017**
	SBP	Complete	0.002	0.001 to 0.003	**0.002**
		Healthy controls	0	−0.001 to 0.002	0.745
		Diabetic patients	0.002	0.001 to 0.004	**0.008**
	BMI	Complete	0.005	0 to 0.009	**0.041**
		Healthy controls	0.009	0.001 to 0.016	**0.020**
		Diabetic patients	0.003	−0.002 to 0.009	0.258
	Waist:hip ratio	Complete	0.793	0.498 to 1.089	**< 0.001**
		Healthy controls	0.472	0.104 to 0.841	**0.012**
		Diabetic patients	0.914	0.456 to 1.371	**< 0.001**
	FBG	Complete	0.019	0.010 to 0.028	**< 0.001**
		Healthy control	0.096	0.023 to 0.169	**0.010**
		Diabetic patients	0.016	0.005 to 0.027	**0.005**
	TGL	Complete	0.044	0.020 to 0.068	**< 0.001**
		Healthy control	0.038	0 to 0.075	**0.049**
		Diabetic patients	0.038	0.007 to 0.070	**0.018**
	TC:HDL	Complete	0.026	0.013 to 0.040	**< 0.001**
		Healthy control	0.021	0.001 to 0.040	**0.037**
		Diabetic patients	0.024	0.006 to 0.043	**0.011**

## Data Availability

All data supporting the findings of this study are available within the paper.
